# Cardiovascular Manifestations in Inflammatory Bowel Disease: A Systematic Review of the Pathogenesis and Management of Pericarditis

**DOI:** 10.7759/cureus.14010

**Published:** 2021-03-20

**Authors:** Ravi S Patel, Sai Rohit Reddy, Adiona Llukmani, Ayat Hashim, Dana R Haddad, Dutt S Patel, Farrukh Ahmad, Domonick K Gordon

**Affiliations:** 1 Internal Medicine, California Institute of Behavioral Neurosciences & Psychology, Fairfield, USA; 2 Behavioral Neurosciences and Psychology, California Institute of Behavioral Neurosciences & Psychology, Fairfield, USA; 3 Plastic and Reconstructive Surgery, California Institute of Behavioral Neurosciences & Psychology, Fairfield, USA; 4 Emergency Department, California Institute of Behavioral Neurosciences & Psychology, Fairfield, USA

**Keywords:** inflammatory bowel disease, pericarditis, extraintestinal manifestations, crohn’s disease, ulcerative colitis

## Abstract

Inflammatory bowel disease (IBD) is a chronic condition of the bowel that can be further categorized into ulcerative colitis and Crohn’s disease. Rarely, this condition can be associated with pericarditis, which can be an extraintestinal manifestation of the disease or drug-induced. This review aims to determine the pathogenesis and management of pericarditis in IBD. In this review, the goal is to elucidate the pathogenesis of pericarditis in IBD and determine if pericarditis is an extraintestinal manifestation of IBD or a complication of current drug therapy used to manage IBD. Additionally, this review intends to explain the first-line management of pericarditis in IBD and explore the role of biologicals in attenuating pericarditis. An electronic search was conducted to identify relevant reports of pericarditis in IBD, and a quality assessment was conducted to identify high-quality articles according to the inclusion criteria. Full-text articles from inception to November 2020 were included, while non-English articles, gray literature, and animal studies were excluded. The majority of studies suggest that pericarditis arises as a complication of drug therapy by 5-aminosalicylic acid derivatives such as sulfasalazine, mesalamine, and balsalazide, and it occurs due to IgE-mediated allergic reactions, direct cardiac toxicity, cell-mediated hypersensitivity reactions, and humoral antibody response to therapy. Drug cessation or the initiation of a corticosteroid regimen seems to be the most effective means of managing pericarditis in IBD due to drug therapy or an extraintestinal manifestation.

## Introduction and background

Over 1.5 million Americans currently live with inflammatory bowel disease (IBD) [1]. IBD is a chronic gastrointestinal tract condition that includes either ulcerative colitis or Crohn’s disease. Ulcerative colitis involves mucosal inflammation that primarily involves the colon, while Crohn’s disease involves transmural inflammation that can manifest as skip lesions throughout the gastrointestinal tract [2]. IBD has equal gender predominance that usually affects young adults less than 30 years old or adults over 50 years of age [3]. Besides gastrointestinal involvement, IBD is associated with various extraintestinal manifestations that commonly involve the musculoskeletal, dermatologic, hepatic, pancreatic, biliary, ocular, renal, and pulmonary systems [4]. Although rare, extraintestinal manifestations involving the heart have been reported, including pericarditis, myocarditis, arrhythmia, and heart failure [5]. Pericarditis is the most common of cardiovascular manifestations, comprising 70% of all cardiovascular complications [5,6].

Currently, the pathogenesis of pericarditis in IBD is unclear. Some authors are unaware of the mechanism by which pericarditis arises, while others speculate or briefly outline the pathogenesis. These include immune-mediated pericarditis, drug-induced hypersensitivity reactions, and cardiotoxicity due to drug treatment [7-10]. Additionally, various treatment modalities are available for the management of extraintestinal pericarditis. However, a consensus on managing this condition has not been met, and various patient outcomes have been reported. 5-Aminosalicylic acid (5-ASA) derivatives, corticosteroids, and non-steroidal anti-inflammatory drugs (NSAIDs) are among the various options that physicians have been prescribing currently [6-9]. As of now, there are various theories of how pericarditis arises in IBD. By understanding the pathogenesis in which pericarditis arises, we can better understand the mechanism of extraintestinal cardiac conditions and prevent them in the management of future patients. If a standard drug regimen is established to treat pericarditis in IBD, unnecessary drug complications and delay in effective treatment can be avoided, and outcomes in future patients can be improved.

In this systematic review, the goal is to elucidate the pathogenesis of pericarditis in IBD and determine if pericarditis is an extraintestinal manifestation of IBD or a complication of current drug therapy used to manage IBD. Additionally, this review intends to explain the first-line management of pericarditis in IBD and explore the role of biologicals in attenuating pericarditis.

## Review

Methods

The following systematic review was conducted as per the Preferred Reporting Items for Systematic Reviews and Meta-Analyses guidelines. The search strategy included an electronic search through the PubMed database by two different authors. Keywords used were “Pericarditis and Inflammatory Bowel Disease,” “Pericarditis and Ulcerative Colitis,” and “Pericarditis and Crohn’s Disease.” Additionally, the following MeSH terms were used: (“Pericarditis”[Mesh]) AND “Inflammatory Bowel Diseases”[Mesh], (“Pericarditis”[Mesh]) AND “Colitis, Ulcerative”[Mesh], (“Pericarditis”[Mesh]) AND “Crohn Disease”[Mesh]. For each keyword or MeSH term, the number of hits on PubMed were noted. Each article was screened based on a title review initially by both authors. All articles included based on title review were further evaluated and either included or excluded for relevancy based on an abstract review. Following the abstract review, a complete article review was done to exclude any other irrelevant articles. Any duplicate articles that coincided with multiple key terms or MeSH terms were also excluded. Any disagreement was resolved with discussion.

Multiple quality assessment tools were utilized to assess the selected articles. The Joanna Briggs Institute checklist was used for case report critical appraisal, while the Scale for the Quality Assessment of Narrative Review Articles checklist was used to assess narrative review articles and letters to the editor. Additionally, the Newcastle-Ottawa Scale was used to assess the quality of cross-sectional studies. For all the scales, a cut-off value of greater than or equal to seven was assigned to designate the articles included in this study.

The eligibility criteria were defined following the Patient, Intervention, Comparison, Outcome approach. The inclusion criteria include all study types and designs from inception to the present day that are related to the topic of pericarditis in IBD. All population groups were included in this study. Only full-text articles were used, and any gray literature was excluded. Non-English articles and animal studies were also excluded from this study. Table 1 and Table 2 show the keywords and MeSH terms used, respectively.

**Table 1:** Regular keywords used in the data search and the number of results.

Regular keywords	Database used	Number of papers
Pericarditis and inflammatory bowel disease	PubMed	90
Pericarditis and ulcerative colitis	PubMed	60
Pericarditis and Crohn's disease	PubMed	35

**Table 2:** MeSH keywords used in the data search and the number of results.

MeSH keywords	Database used	Number of papers
(“Pericarditis”[Mesh]) AND “Inflammatory Bowel Diseases”[Mesh]	PubMed	69
(“Pericarditis”[Mesh]) AND “Colitis, Ulcerative”[Mesh]	PubMed	43
(“Pericarditis”[Mesh]) AND “Crohn Disease”[Mesh]	PubMed	25

Results

Search Results

Initial screening of the electronic database PubMed yielded 322 records. Of these, 226 were duplicates. Of the 96 records that were relevant, 31 were excluded based on the relevancy of title and abstract review. A total of 65 records underwent full title review, of which 15 were excluded due to irrelevancy. The remaining 50 articles underwent quality assessment, of which 11 articles were excluded as the score was less than seven. According to the inclusion criteria, 39 total articles were analyzed in this qualitative study.

Study Characteristics

This analysis included 36 patients, of whom 12 were female and 24 were male. The patients’ age ranged from nine to 76 years, with an average age of 30.8 years. Of the patients with IBD, 11 had Crohn’s disease and 25 had ulcerative colitis. Geographically, 19 studies were from the United States, 12 studies were from Europe, three studies were from Canada, three studies were from Japan, one study was from Israel, and one study was from Turkey.

Study Quality

Of the 50 articles that underwent quality assessment, 39 were considered high quality with a score of greater than or equal to seven. A total of 11 articles were excluded due to a score of less than seven. Of the 39 included studies, 22 had a quality assessment score of 8/8, and 17 studies had a score of 7/8.

Actual Results

**Figure 1:**
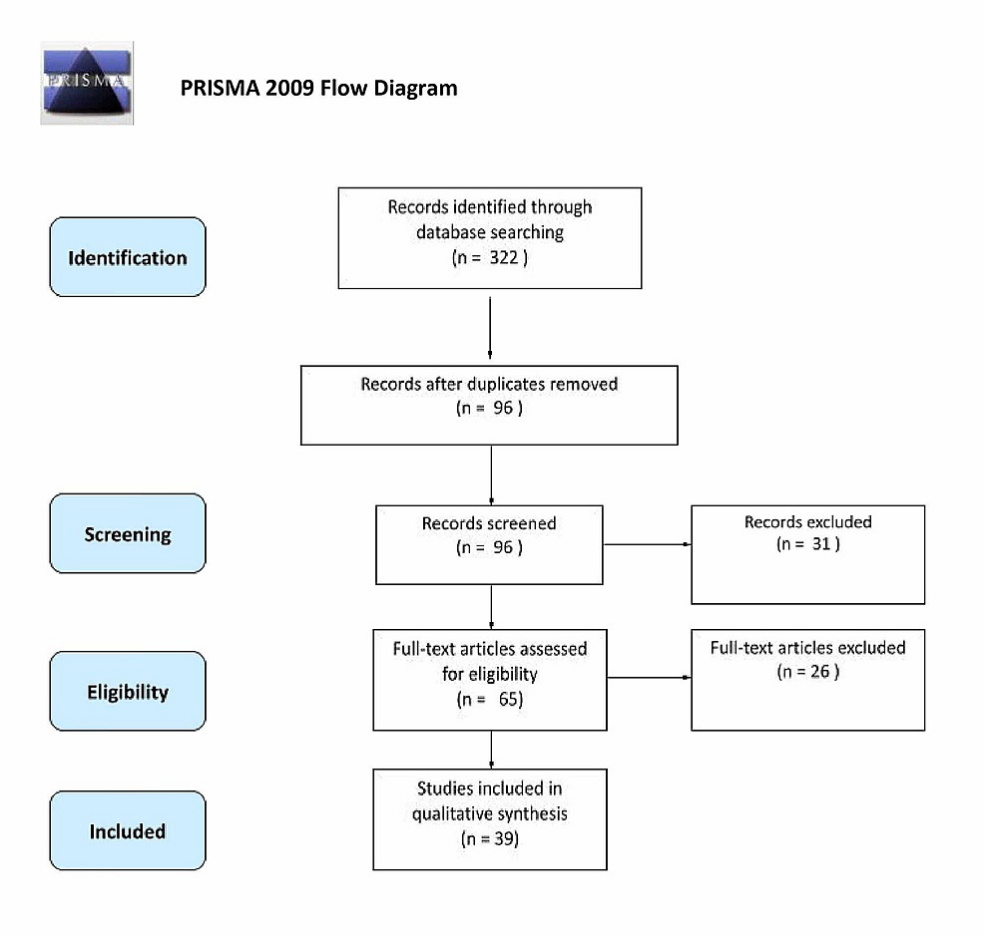
PRISMA flow diagram showing the data selection process. PRISMA, Preferred Reporting Items for Systematic Reviews and Meta-Analyses

A total of 16 studies offered information related to the pathogenesis of pericarditis arising in IBD. The majority of studies suggest that pericarditis arises from IBD complications due to drug therapy by 5-ASA derivatives such as sulfasalazine, mesalamine, and balsalazide. Additionally, drug-induced pericarditis in patients with IBD has been reported with infliximab and azathioprine therapy. Proposed mechanisms include IgE-mediated allergic reactions by 5-ASA drugs, direct cardiac toxicity induced by medical treatment, cell-mediated hypersensitivity reactions due to therapy, and humoral antibody response to therapy. Additionally, drug-induced lupus reactions and serum sickness-like reactions have been suggested. A total of 38 studies provided information related to the management of pericarditis in IBD patients. Nine cases of pericarditis resolved with drug cessation alone, and nine cases required drug cessation along with corticosteroid treatment. Eight cases only required steroid therapy to resolve the condition. Five cases required drug cessation and NSAID use, while two cases resolved solely with colectomy. Three patients developed a pericardial tamponade that resolved upon pericardiocentesis. In two cases, mesalamine drug treatment was stopped and replaced with either azathioprine or infliximab to resolve the patient’s pericarditis. Figure 1 illustrates the process of identifying relevant studies.

Discussion

Pathogenesis

Interpretation: In most studies included in this review, pericarditis occurred after drug therapy was initiated to treat either Crohn’s disease or ulcerative colitis. Out of the 36 patients included in this study, only four presented with pericarditis that was not induced by drug therapy [11-14]. Although a rare complication, this suggests that pericarditis arises more often as an adverse drug reaction rather than as an extraintestinal manifestation of the disease course itself. Of the drug-induced cases, 5-ASA derivatives such as sulfasalazine, mesalamine, and balsalazide were responsible for pericarditis in most patients.

Analysis: Pericarditis arising due to 5-ASA derivatives have various pathological mechanisms. Mitchell et al. and Bernardo et al. suggest that an IgE-mediated hypersensitivity reaction may be responsible [15,16]. Direct cardiotoxicity has also been proposed where 5-ASA derivatives directly damage myocytes, exposing antigens and releasing inflammatory mediators, with the latter eliciting an immune response [17]. Cell-mediated hypersensitivity and humoral antibody responses to 5-ASA derivatives have also been suggested. The humoral antibody theory, where antibodies are generated against pericardial antigens due to 5-ASA drug exposure, is considered to be one of the most plausible explanations. Owing to sulfasalazine or mesalamine intake, antibodies are generated against pericardial antigens leading to inflammation [16-21]. Patients taking sulfasalazine who develop pericarditis are thought to have developed a lupus-like reaction [22-24]. This is supported by lab results seen in patients with sulfasalazine-induced pericarditis. Patients present with positive antinuclear antibodies (ANA), elevated erythrocyte sedimentation rate (ESR), fevers, arthralgia, and arthritis months to years after starting treatment, which suggest lupus-like reaction. Mesalamine is thought to induce a type IV drug-induced hypersensitivity reaction, which is supported by ANA +/-, ESR raised +/-, lymphocyte stimulation test +, with a resolution of pericarditis on drug cessation and initiation of a steroid regimen [23,25]. When pericarditis arises solely as an extraintestinal manifestation of IBD, lab reports usually show ANA - and ESR raised -. However, most cases in this study had ANA +, elevated ESR, and drug history of a 5-ASA derivative, which suggests that pericarditis is more likely to arise due to drug therapy rather than disease progression in IBD. In our study of 18 cases, drug cessation with or without initiating a steroid regimen was effective at resolving the patients’ condition.

Additionally, Ishikawa et al. and Coman et al. have presented cases where pericarditis arose after 5-ASA therapy, resolved upon drug cessation, and reoccurred on reinitiation of the causative drug [25,26]. Patients on 5-ASA therapy for the treatment of pericarditis usually develop symptoms within two weeks of treatment initiation, which resolves upon drug cessation and initiation of a steroid regimen [23]. This further suggests that drug therapy is more likely to lead to pericarditis versus an extraintestinal manifestation of IBD. In two cases, infliximab was suspected of causing pericarditis in IBD patients. Two different mechanisms have been proposed. Burke et al. suggests that infliximab can cause a drug-induced SLE reaction leading to various inflammatory manifestations such as pericarditis [27]. On the other hand, Devasahayam et al. suggests that infliximab therapy has a pro-inflammatory effect on the pericardium leading to a serum sickness-like reaction. This is supported by the fact that patients may develop fever, rash, myalgia, and polyarthralgia one to two weeks after initiating therapy [28]. One patient also developed pericarditis while on azathioprine therapy. Adverse reactions due to azathioprine have been known to be caused by a hypersensitivity reaction [29]. This patient developed pericarditis within one to two weeks after azathioprine was initiated, and his condition resolved once the drug was stopped. Azathioprine-induced hypersensitivity is more likely to be responsible for the development of pericarditis versus an extraintestinal manifestation because rechallenge causes symptoms to develop more rapidly than the initial one to two weeks of using the drug [29]. However, one case pointed more towards an extraintestinal manifestation of pericarditis. Perrot explains that when sulfasalazine was given to a patient, pericarditis developed two weeks later. After drug cessation, pericarditis resolved. To maintain remission, sulfasalazine was given once more, but pericarditis did not recur. Although uncertain, pericarditis may not have recurred as it may have arisen as an extraintestinal manifestation or possibly due to a lowered sulfasalazine dose to maintain remission [30]. Table 3 lists the intervention given during each study and the pathogenesis of pericarditis in IBD.

**Table 3:** Detailing the results and pathogenesis of pericarditis in IBD. IBD, inflammatory bowel disease; 5-ASA, 5-aminosalicylic acid; UC, ulcerative colitis; NSAIDs, non-steroidal anti inflammatory drugs; EIM, extraintestinal manifestations; WBCs, white blood cells

Author	Year of publication	Type of study	Result (intervention given)	Conclusion
Bunu et al. [17]	2019	Clinical review	No specific intervention mentioned	Pericarditis can be caused by immune-mediated myocarditis in IBD as a result of exposure to autoantigens or cardiotoxicity as an adverse effect of the treatment with 5-ASA and its derivatives
Mitchell et al. [15]	2018	Clinical review	No specific intervention mentioned	Aminosalicylate therapy leads to IgE-mediated allergic reactions, direct cardiac toxicity, cell-mediated hypersensitivity, or a humoral antibody response against 5-ASA derivatives
Dipasquale et al. [31]	2017	Case report	Infliximab was given to treat IBD.Pericarditis occurred and was managed with steroids	Infliximab-induced pericarditis can occur through the following mechanisms: direct cardiac toxicity, IgE-mediated allergic reaction, humoral antibody response, cell-mediated hypersensitivity, or serum sickness-like reaction, and drug-induced lupus
Bernardo et al. [16]	2016	Case report	Mesalamine therapy stopped after pericarditis occurred. Steroids and azathioprine therapy started to resolve pericarditis	Pericarditis arises due to: IgE-mediated allergic reaction, direct cardiac toxicity, cell-mediated hypersensitivity, or a humoral antibody response
Coman et al. [25]	2014	Clinical review	Balsalazide given with mesalamine which lead to pericarditis. Cessation of both drugs rapidly resolved the condition	Balsalazide causes a drug-induced hypersensitivity reaction
Nair et al. [21]	2014	Case report	Mesalamine therapy lead to pericarditis and resolved on drug cessation	Mesalamine leads to a humoral-mediated hypersensitivity reaction where antibodies are generated against cardiac antigens
Sonu et al. [19]	2013	Case report	Patient on mesalamine and sulfasalazine therapy developed pericarditis Both drugs were stopped, and pericarditis resolved. On initiation of another 5-ASA derivative, balsalazide, pericarditis recurred and was more severe. Balsalazide cessation resolved pericarditis	A patient who develops pericarditis on 5-ASA derivatives may have a more severe reaction on replacement with another derivative. Immediate cessation of 5-ASA derivatives in both instances of myopericarditis suggests that there is a drug-induced hypersensitivity reaction
Burke et al. [27]	2007	Letter to the editor	Infliximab caused lupus-like symptoms, including pericarditis. Drug cessation resolved pericarditis	Infliximab can cause a drug-induced SLE reaction leading to various inflammatory manifestations such as pericarditis
Devasahayam et al. [28]	2007	Letter to the editor	Infliximab therapy lead to pericarditis. Infliximab discontinued, and NSAIDS given to resolve the condition	Infliximab may have pro-inflammatory activity in certain tissues, including the pericardium leading to a serum sickness-like reaction
Oxentenko et al. [20]	2002	Case report	Mesalamine given initially leading to pericarditis. Steroids were given to resolve pericarditis	Mesalamine can lead to pericarditis due to a direct cardiotoxic effect, cell-mediated hypersensitivity reaction, IgE-mediated allergic reaction, or a humoral antibody response. Most patients with mesalamine-induced pericarditis have presented within two weeks of initiating the drug
Mahajan et al. [22]	2001	Case report	Mesalamine treatment was initiated and then stopped after pericarditis occurred. Methylprednisolone was then given, which resolved pericarditis	Mesalamine may lead to a hypersensitivity reaction. Sulfasalazine can cause a lupus-like reaction
Vayre et al. [24]	1998	Letter to the editor	Patient admitted with pericarditis and IBD. Mesalamine cessation resolved pericarditis	Sulfasalazine leads to a lupus-like reaction causing pericarditis
Granot et al. [32]	1988	Clinical review	Aspirin was given to resolve pericarditis	5-ASA may inhibit prostaglandin function and metabolism and may also disrupt polymorphonuclear WBCs

Management

Interpretation: Most patients responded well to drug cessation with or without initiation of corticosteroid treatment. Additionally, a handful of patients responded well to steroids alone. This accounted for 26 of the 36 patients, suggesting the efficacy of drug cessation and corticosteroid therapy as the first-line management of pericarditis in IBD. NSAID therapy, colectomy, pericardiocentesis, and initiation of alternative therapies to 5-ASA derivatives (e.g., azathioprine, infliximab) comprised the management of the remaining 10 patients, suggesting a less preferred or alternative management if drug cessation and corticosteroids are ineffective.

Analysis: As pericarditis is likely to occur due to a drug-induced hypersensitivity reaction, many pericarditis cases are resolved simply by drug cessation. Obtaining a thorough patient history and a past and present drug history is crucial to identify the etiology of the patient’s condition, which may be managed by changing the drug therapy. Most patients are advised to discontinue aminosalicylate therapy and are given a steroid regimen, which allows the condition to resolve within two weeks [15]. Several studies suggest that solely giving high-dose corticosteroids is adequate to resolve pericarditis [12,14,20,33-37]. However, Dias et al. suggests that the efficacy of corticosteroids is uncertain because the time of resolution of pericarditis with steroids is similar to the time of resolution by simply stopping drug therapy [38]. Sposato further supports this idea as corticosteroids alone were not sufficient to resolve their patient’s pericarditis. It was only until mesalazine therapy was withdrawn that their patient began to recover [39]. This suggests that when managing a patient with pericarditis in IBD, known causative drugs should be immediately stopped, and a corticosteroid regimen can be then considered. However, if pericarditis arises as an extraintestinal manifestation unrelated to drug therapy, corticosteroids are effective and preferred to manage the condition [40]. While tapering doses of steroids, it is essential to monitor for recurring pericarditis; if pericarditis recurs, the dose of steroids given should be increased [11]. Furthermore, it is essential to rule out any infectious etiology or contraindications to immunosuppressive therapy before initiating steroids. NSAIDs such as aspirin or indomethacin are other alternative treatments for managing pericarditis in IBD [17,18,32,40,41]. The treatment of IBD-induced pericarditis is steroids in 80% of the cases. The remainder of cases can be managed with aspirin or indomethacin, in which pericarditis responds well. Aspirin and indomethacin can be preferred before giving steroids as long as IBD is dormant. Although effective at managing pericarditis, both aspirin and indomethacin can exacerbate a patient’s IBD. In active bowel disease, selective COX-2 inhibitors such as celecoxib can be given [17,20]. Colchicine is another alternative drug that can be given, although active bowel disease should not be present as it may lead to exacerbation of diarrhea [17]. In two cases, azathioprine and infliximab were given to replace mesalamine therapy. Although pericarditis did not recur, these drugs are not recommended as they are responsible for leading to pericarditis in several other cases [31,33]. In three cases, a colectomy was done. However, steroids were also initiated in two of the three cases; hence, the efficacy of colectomy alone is difficult to ascertain [42-44]. As a complication of pericarditis, pericardial effusion and pericardial tamponade may arise. Drainage by pericardiocentesis is effective at resolving the effusion. Alternatively, pericardiectomy can be done if pericarditis complications arise [11,12,41,44].

It is difficult to distinguish that a patient who has developed pericarditis is due to an extraintestinal manifestation of IBD disease progression or an adverse effect of drug therapy. Thorough patient history and careful monitoring of the patient’s condition upon drug removal and initiation are needed to manage the patient better. There is also uncertainty about the use of infliximab and azathioprine therapy in managing pericarditis with IBD. Although some cases were resolved with such therapy, others were incited by the same. The risk factors leading to pericarditis in IBD are unclear. Understanding the risk factors and etiology can help in the prevention and immediate management of pericarditis in IBD. Table 4 shows the intervention given for each study and the management of pericarditis in IBD.

**Table 4:** Detailing the intervention and management of pericarditis in IBD. IBD, inflammatory bowel disease; 5-ASA, 5-aminosalicylic acid; UC, ulcerative colitis; NSAIDs, non-steroidal anti-inflammatory drugs; EIM, extraintestinal manifestations

Author	Year of publication	Type of study	Result (intervention given)	Conclusion
Bunu et al. [17]	2019	Clinical review	No specific intervention was given	NSAIDs can be given to treat pericarditis. Selective COX-2 inhibitors are preferred to avoid gastrointestinal toxicity. Colchicine is an option for therapy but causes diarrhea. Immunosuppressives (corticosteroids, azathioprine, cyclosporine) is another option, but you must rule out an infectious etiology first
Mitchell et al. [15]	2018	Clinical review	No specific intervention was given	The majority of patients should discontinue aminosalicylates and give steroids, which leads to resolution within two weeks. Aspirin or colchicine must be used with caution because it has gastrointestinal side effects. Infliximab and azathioprine may induce pericarditis when treating IBD. Pericarditis can arise as an extraintestinal manifestation outside of drug induction
Dias et al. [38]	2018	Case report	UC treated with mesalazine and pericarditis developed later and resolved after drug cessation. Pericarditis recurred once mesalazine therapy continued	5-ASA drug cessation is adequate to treat pericarditis with IBD. Efficacy of corticosteroids is uncertain because the time of resolution of pericarditis with steroids is similar to just stopping drug therapy
Bernardo et al. [16]	2016	Case report	Mesalamine therapy stopped after pericarditis arose. Steroids and azathioprine therapy resolved pericarditis	Clinical manifestations occur 2-4 weeks of mesalamine treatment. Stopping mesalamine resolves pericarditis within 7-14 days. Reintroducing mesalamine leads to recurrent pericarditis. Changing the route of administration (oral to enema) for mesalamine may lead to pericarditis
Kiyomatsu et al. [45]	2015	Case report	Mesalamine induced pericarditis occurred and resolved on drug cessation. Infliximab therapy was used to replace mesalamine	Drug cessation of mesalamine is adequate to resolve pericarditis in IBD. Pericarditis can arise as an EIM or due to drug therapy for IBD
Nair et al. [21]	2014	Case report	Mesalamine initiated, and pericarditis developed. It resolved on drug cessation	Treatment includes drug cessation, supportive care, and monitoring of the patient. It is important to take a proper patient history, including past and present drug therapy, and carry out lab tests to differentiate EIM or drug-induced pericarditis
Sonu et al. [19]	2013	Case report	A patient on mesalamine and sulfasalazine developed pericarditis. When both drugs stopped, pericarditis resolved. On initiation of another 5-ASA derivative, balsalazide, pericarditis recurred and was more severe. Balsalazide cessation resolved the pericarditis	A patient who developed pericarditis on 5-ASA derivatives may have a more severe reaction if therapy is replaced with an alternative derivative. Immediate cessation of 5-ASA derivatives in both instances of myopericarditis suggests that there is a drug-induced hypersensitivity reaction
Abu-Hijleh et al. [33]	2010	Case report	Mesalamine and azathioprine were given to the patient and pericardial effusion developed. The patient was given prednisolone and effusion resolved. On tapering the steroid dose, pericarditis recurred	Steroids are effective at treating IBD with pericarditis and pericardial effusion
Sposato et al. [39]	2010	Case report	Mesalazine was given, stopped, and corticosteroids were added to resolve pericarditis	Corticosteroids are not enough to resolve pericarditis. Mesalazine treatment must also be stopped due to direct drug toxicity as evidence shows mesalazine and steroids together still did not resolve pericarditis. Cessation of mesalazine alone can be effective
Cappell et al. [11]	2008	Case report	Mesalamine treatment stopped eight years before pericarditis occurred, and lab studies suggest pericarditis arose as an EIM. Indomethacin initially given for pericarditis, but UC recurred. Steroids were given to resolve both IBD and pericarditis	Prednisolone should be preferred over NSAIDS as it can treat both IBD and pericarditis, while NSAIDS only treat the latter and exacerbate the former. While tapering doses of steroids, always check for recurring pericarditis; if present, increase dose of steroid. Pericardiectomy can be done to resolve life-threatening cardiac tamponade
Perrot et al. [30]	2007	Case report	Sulfasalazine given, and pericarditis occurred. Drug therapy was stopped, and pericarditis resolved. On reinitiation of therapy, pericarditis did not recur	IBD causes pericarditis if bowel disease is active, and pericarditis is not solely drug-induced
Jackson et al. [40]	2005	Letter to the editor	Corticosteroids were given to resolve pericarditis	In non-medication-induced pericarditis, corticosteroids are effective at resolving the condition. NSAIDs are also useful but should be avoided in active bowel disease as they can worsen the condition
Hyttinnen et al. [13]	2003	Case report	Immunosuppressive therapy given on the first three episodes of IBD. No mesalamine was given until the fifth episode occurred. Pericarditis developed in the first three episodes without mesalamine	Pericarditis can arise as an EIM not related to 5-ASA derivatives
Oxentenko et al. [20]	2002	Case report	Mesalamine was given to treat IBD and pericarditis occurred. Mesalamine was discontinued, and steroids resolved pericarditis	Treatment of IBD-induced pericarditis is steroids in 80% of cases. Remainder of cases can be managed with aspirin or indomethacin. Pericarditis responds well to aspirin and indomethacin and can be preferred before giving steroids as long as IBD is dormant
Dubowitz et al. [43]	2001	Case report	No 5-ASA given (patient was intolerant). Prednisolone and subtotal colectomy was done. Pericarditis then manifested with effusion later and pericardiocentesis was done to resolve the condition with the continuation of steroids (steroids alone were not sufficient)	Pericarditis can arise as an extraintestinal manifestation not related to drug use. In the case of pericardial effusion, pericardiocentesis is needed
Orii et al. [37]	2001	Case report	The patient was given salazosulfapyridine and pericarditis occurred. Pericarditis resolved with steroids	Corticosteroids helped resolve pericarditis
Molnar et al. [36]	1999	Case report	Sulfasalazine given to treat IBD, then discontinued. Methylprednisolone given to manage IBD and pericarditis	Pericarditis resolved with corticosteroids
Gujral et al. [18]	1996	Case report	Mesalamine therapy caused pericarditis. Mesalamine then discontinued. Aspirin given, which resolved pericarditis	Drug cessation and NSAIDS can be given to manage pericarditis in IBD
Sarrouj et al. [35]	1994	Case report	Indomethacin therapy given initially without resolution of pericarditis. IV methylprednisolone was given and aspirin then initiated later, which led to resolution.	Corticosteroids are effective in treating pericarditis
Granot et al. [32]	1988	Clinical review	5-Aminosalicylates led to pericarditis. Aspirin given in tapering doses to resolve condition	Aspirin may help resolve pericarditis, but most cases respond to corticosteroids
Farley et al. [41]	1986	Case report	Sulfasalazine was continuously given throughout the management of the patient. Steroids were then given, which resolved the pericarditis. A rapid drop in tapered steroid doses caused pericarditis to recur	Steroids are effective at treating pericarditis. NSAIDs can be used as an alternative treatment. Pericardiecetomy can be done to resolve cardiac tamponade. Gradual tapering of steroid therapy is needed. Recurrence can be treated by introducing or increasing the dose of steroids or alternatively using NSAIDs
Manomohan et al. [34]	1984	Case report	IV steroids and parenteral nutrition was given to manage IBD and pericarditis	High-dose corticosteroids are adequate enough for treating pericarditis
Levin et al. [42]	1979	Case report	Colectomy resolved both IBD and pericarditis. Prednisolone was given after	Colectomy can be curative for both diseases
Rheingold [44]	1975	Letter to the editor	Colectomy was done to treat IBD and pericarditis arose after colectomy. It was treated with pericardial drainage and no steroids given	Cardiac tamponade can frequently arise following pericarditis. Usually, pericarditis resolves after colectomy. Effusion can be effectively treated with drainage
Breitenstein et al. [12]	1974	Letter to the editor	Pericarditis arose as an EIM. Steroid treatment resolved the pericarditis. Pericardiocentesis was done to resolve the pericardial tamponade	Steroids are effective at treating IBD with pericarditis. Cardiac tamponade can be managed with pericardiocentesis

Limitations

This study’s limitations include the limited availability of clinical reviews, systematic reviews, cohort, and case-control studies. Although some clinical reviews and systematic reviews were included, most studies were case reports and letters to the editor. Additionally, non-English papers were excluded, and animal studies were not used in this review. The studies used in this review included all available studies from the inception to the present rather than only recent studies. Full-text articles, if not available, were also not included in this study. The studies that were excluded or not available could have provided relevant information about the pathogenesis or management of pericarditis in IBD and improved the overall results.

## Conclusions

Cardiovascular manifestations of IBD, particularly pericarditis, is an uncommon occurrence in which the pathogenesis and management have previously not been explored in detail. Pericarditis in IBD can arise due to drug therapy or, less commonly, as an extraintestinal manifestation. 5-ASA derivatives are primarily responsible for inducing pericarditis through IgE-mediated hypersensitivity reactions, direct cardiotoxicity, cell-mediated hypersensitivity, and humoral antibody reactions. Infliximab and azathioprine are less common causes of pericarditis and are most likely caused by a lupus-like reaction and drug-induced hypersensitivity, respectively, making them less suitable options when trying to replace 5-ASA drug therapy. Most patients respond well to drug cessation and corticosteroid therapy. Aspirin, indomethacin, and colchicine can be used as an alternative therapy as long as bowel disease is not active, and drainage may be required in the case of effusion or cardiac tamponade. Additional studies should be conducted to identify how to differentiate drug-induced pericarditis from extraintestinal manifestations of IBD. Additionally, the risk factors associated with developing pericarditis in an IBD patient should be studied to help prevent and manage this condition.
